# Apgar score and risk of autism

**DOI:** 10.1007/s10654-018-0445-1

**Published:** 2018-10-05

**Authors:** Amirhossein Modabbernia, Sven Sandin, Raz Gross, Helen Leonard, Mika Gissler, Erik T. Parner, Richard Francis, Kim Carter, Michaeline Bresnahan, Diana Schendel, Mady Hornig, Abraham Reichenberg

**Affiliations:** 1 Department of Psychiatry and Seaver Autism Center, Icahn School of Medicine at Mount Sinai, 1 Gustave L Levy PLC, New York, NY 10029 USA; 2 Seaver Autism Center, Icahn School of Medicine at Mount Sinai, New York, USA; 3 Department of Medical Epidemiology and Biostatistics, Karolinska Institutet, Stockholm, Sweden; 4 Department of Epidemiology and Preventive Medicine, Tel Aviv University, Tel Aviv, Israel; 5 Division of Psychiatry, Chaim Sheba Medical Center, Tel Hashomer, Israel; 6 Telethon Kids Institute, University of Western Australia, Crawley, Australia; 7 National Institute for Health and Welfare, Helsinki, Finland; 8 Nordic School of Public Health, Gothenburg, Sweden; 9 Department of Child Psychiatry, Turku University and Turku University Hospital, Turku, Finland; 10 Section for Biostatistics, Department of Public Health, Aarhus University, Aarhus, Denmark; 11 Department of Epidemiology, Mailman School of Public Health, Columbia University, New York, NY USA; 12 New York State Psychiatric Institute, New York, NY USA; 13 Department of Public Health, Institute of Epidemiology and Social Medicine, Aarhus University, Aarhus, Denmark; 14 Department of Economics and Business, National Centre for Register-Based Research, Aarhus University, Aarhus, Denmark; 15 Lundbeck Foundation Initiative for Integrative Psychiatric Research, iPSYCH, Aarhus, Denmark; 16 Center for Infection and Immunity, Mailman School of Public Health, Columbia University, New York, NY USA; 17 Department of Preventive Medicine, Icahn School of Medicine at Mount Sinai, New York, USA; 18 Friedman Brain Institute, Icahn School of Medicine at Mount Sinai, New York, USA

**Keywords:** Epidemiology, Autism, Apgar score, Etiology

## Abstract

Low Apgar score has been associated with higher risk for several neurological and psychiatric disorders, including cerebral palsy and intellectual disability. Studies of the association between Apgar score and autism spectrum disorder (ASD) have been inconsistent. We aimed to investigate (1) the association between low Apgar score at 5 min and risk for ASD, and (2) the modifying effects of gestational age and sex on this association in the largest multinational database of ASD. We included prospective data from 5.5 million individuals and over 33,000 cases of ASD from Norway, Sweden, Denmark and Western Australia who were born between 1984 and 2007. We calculated crude and adjusted risk ratios (RR) with 95% confidence intervals (95% CIs) for the associations between low Apgar score and ASD. All analyses for ASD were repeated for autistic disorder (AD). We used interaction terms and stratified analysis to investigate the effects of sex, gestational age, and birth weight on the association. In fully adjusted models, low Apgar scores (1–3) (RR, 1.42; 95% CI, 1.16–1.74), and intermediate Apgar scores (4–6) (RR, 1.50; 95% CI, 1.36–1.65) were associated with a higher RR of ASD than optimal Apgar score (7–10). The point estimates for low (RR, 1.88; 95% CI, 1.41–2.51) and intermediate Apgar score (RR, 1.54; 95% CI, 1.32–1.81) were larger for AD than for ASD. This study suggests that low Apgar score is associated with higher risk of ASD, and in particular AD. We did not observe any major modifying effects of gestational age and sex, although there seems to be substantial confounding by gestational age and birth weight on the observed association.

**Electronic supplementary material:**

The online version of this article (10.1007/s10654-018-0445-1) contains supplementary material, which is available to authorized users.

## Introduction

The etiology of autism spectrum disorder (ASD) is still poorly understood. Evidence implies a strong genetic component for ASD [1], but a key role for the environment and its interaction with genetic factors has also been suggested [2, 3]. Several population-based studies and subsequent meta-analyses suggest that birth complications are associated with an increased risk of ASD [4–9].

Apgar score is a common tool for assessment of health status in the immediate neonatal period [10]. This measure assesses the neonatal well-being status based on heart rate, respiration, color, muscle tone, and reflex irritability after delivery. It is assessed ubiquitously in every newborn around the world and therefore can be considered an invaluable, if not optimal source of information about neonatal status immediately after birth. Several studies have shown low Apgar score to be affected by anoxia, infection, prematurity, maternal sedation, or trauma [10–13]. Since its introduction in 1953 by Virginia Apgar, numerous studies have tried to establish the utility of Apgar score for prediction of long-term morbidity and mortality [14]. Despite its overall suboptimal reliability [15], the American Academy of Pediatrics and the American Academy of Obstetricians and Gynecologists in their 2015 statement concluded “monitoring of low Apgar scores from a delivery service may be useful” [10]. Low 5 min Apgar score in particular has been associated with increased risk of early postnatal mortality, cerebral palsy (CP), seizure and mental retardation [4, 16–19]. Studies investigating the association between Apgar score and ASD risk have had mixed results [5–7, 20–24]. In a meta-analysis of six population-based studies with a total of 370,173 individuals, we found a slightly increased risk of ASD (OR, 1.67; 95% CI, 1.34–2.09) in subjects with a 5-min Apgar score < 7 [25]. However, meta-analyses are frequently limited by factors such as statistical heterogeneity, publication bias, and underreporting of null studies. In particular, studies included in our meta-analysis did not examine the association between Apgar score and ASD across the entire range of the Apgar score, and relied on different cut-off scores to define the “low Apgar score”. Moreover, the studies used different designs and varying set of variables as potential covariates/confounders in the adjusted analysis, which makes the pooled estimates difficult to interpret. Because both ASD and low Apgar score (< 4) are rare events, earlier attempts to address this association has also been hampered by limited sample size.

In addition, in previous studies of ASD and Apgar score, effects of potential modifiers like sex and gestational age have not received adequate attention. Both sex and gestational age are important determinants of birth outcomes, and, at the same time, key factors in modifying the risk of ASD [20, 26–29]. Moreover, sex modifies phenotypic characteristics and comorbid condition in patients with ASD [26, 28]. Importantly, in patients with ASD, females are at higher risk of developing comorbid epilepsy and intellectual disability (ID) [26, 28] two conditions that have frequently been associated with low Apgar score [4, 30]. It is therefore important to investigate the modifying effects of gestational age and sex on the association between Apgar score and risk of ASD.

The primary aim of this study was to test for an association between low Apgar score and the risk of ASD. The secondary objective, which was made possible by the uniquely large sample size, was to assess the effect of gestational age, sex, and ASD diagnostic subtype on the relationship between ASD and Apgar score.

## Methods

### Study design

The current study uses birth characteristics and ASD diagnoses from Norway, Sweden, Denmark, and Western Australia available from the International Collaboration for Autism Registry Epidemiology (iCARE) [31]. iCARE combines population based national birth cohorts with essentially complete follow-up of clinical diagnoses of autism. The data in iCARE are provided with ethical approvals for data access as well as waivers for informed consent since all data are register-based and the registered persons were not contacted.

### Study population

The study population comprised all children born from 1984 to 2007 for Sweden, from 1987 to 2007 for Denmark, from 1984 to 2005 for Norway, and from 1984 to 1999 for Western Australia. Children who died before 1 year of age were excluded from the sample.

### Exposure, outcome, and covariates

The primary exposure variable in the present study was the Apgar score at 5 min, which is a more reliable predictor of morbidity and mortality than 1-min Apgar score [10, 17]. This is biologically plausible, because children who show lower scores at 5 min (compared to 1 min) are more likely to have a serious condition that precluded their successful resuscitation [10]. Apgar score was treated as continuous (for testing quadratic term), as individual scores over 10 categories (for calculating individual estimates), or as categorical within categories of 1–3, 4–6, and 7–10 (reference group). The 3 categories (1–3, 4–6, 7–10) were chosen based on the most common categorization found in the scientific literature [11, 17, 25], with the exception that children with Apgar score of zero were not included in the analysis, because reporting of Apgar score of zero was missing in Western Australia, while in Denmark an Apgar score of zero sometimes indicated a transfer to the intensive care unit immediately after birth [30, 32, 33].

Diagnostic information for the Danish and Swedish populations was derived from health registries whereas in the Norwegian and Western Australian populations, it was extracted from government-maintained service/benefits registries. In Denmark, The International Classification of Diseases, Eighth Revision (ICD-8) was used for diagnosing ASD from 1966 to 1993 followed by ICD-10 from 1993 forward. Child psychiatrists were in charge of making the diagnosis of ASD in all cases. ASD diagnosis is not validated in Denmark, but the validity is believed to be generally high [34]. In Norway, Norwegian National Insurance System (NNIS) contains all the diagnosed cases of ASD made by a child psychiatrist or a paediatrician. A validation study by the Norwegian Specialist Health Services whose data is derived from NNIS found that 97% of registered cases met the Diagnostic and Statistical Manual of Mental Disorders, 4th edition (DSM-IV) criteria for ASD [35]. In Sweden diagnosis of ASD is usually made by a child psychiatrist. In a validation study conducted in Stockholm County, 96% of cases of ASD were verified [36]. In Western Australia, for children with ASD to be eligible for early intervention, the diagnosis of ASD needs to be confirmed by a multidisciplinary team. There is no specific validation study of ASD diagnosis in Western Australia. Further details of harmonization of ASD diagnosis along with diagnostic codes, case identification, reliability and registry reporting procedures have been described in detail elsewhere [See Ref. [31] and Appendix B in Ref. [36]].

Data regarding 5 min Apgar score, maternal age, paternal age, gestational age, sex, birth weight, and birth multiplicity were derived from birth or civil registries [31].

Birth year (1984–1988, 1989–1993, 1994–1998, 1999–2003 and 2004–2007) and site (Denmark, Norway, Sweden, Western Australia) were considered as mandatory covariates in all models. Other potentially confounding covariates were maternal age (categories of ≤ 19, 20–24, 25–29, 30–34, 35–39, and ≥ 40 years), paternal age (categories of ≤ 19, 20–24, 25–29, 30–34, 35–39, 40–44, and ≥ 45 years), multiple birth, sex, gestational age (preterm ≤ 36, term 37–40, and post-term ≥ 41 weeks), and birth weight (< 1000, 1000–1499, 1500–2499, 2500–3999, ≥ 4000 g). Gestational age and sex were also considered as potential effect modifiers.

### Statistical analysis

We fitted log binomial regression in generalized linear models to calculate relative risk (RR) and two-sided 95% Wald-type confidence intervals (95% CI) for the association between Apgar score and ASD. We ran four models. In model 1, we adjusted only for site and birth year. We fitted three additional models with increasing degree of control for covariates. In model 2, we adjusted for variables in model 1 along with maternal and paternal age and sex. In model 3 we adjusted for the variables in model 2 plus gestational age. In model 4, we adjusted for the variables in model 3 plus birth weight. All analyses were done on a dataset with complete information for all model covariates.

We calculated RRs (95% CIs) for the associations between ASD and both three-level and ten-level categories of Apgar score. In the analyses of the three-level categories, results were presented for the three groups of low (1–3) and intermediate (4–6) with the optimal Apgar score (7–10) being the reference group. We complemented the analysis using categories of Apgar score with analysing the association between continuous Apgar score and risk of ASD. In the analyses of 10-level categories, results were presented in nine categories with Apgar score of 10 being the reference group. We further examined the shape of the association of individual Apgar scores on a continuous scale by including a quadratic term in the regression models, and performed a statistical test if this term was larger than zero. To rule out the possible effect of multiple gestations on the analyses, we reanalysed the data by restricting the analyses to singleton births only.

To investigate the effect modification by sex and gestational age we conducted subgroup analyses by stratifying by gestational age (≤ 36, 37–40 and ≥ 41 weeks), and sex. To assess the degree of modification by sex and gestational age we compared the RR of ASD in neonates with low versus high Apgar score between male and female offspring, and the RR of ASD in neonates with low versus high Apgar score between preterm, term, and post-term born children. We included an interaction term for Apgar score by sex or Apgar score by gestational age in respective models. In sensitivity analysis, all analyses for ASD were repeated for autistic disorder (AD), the most severe form of ASD often with intellectual disability as co-morbid diagnosis, as the outcome (Supplementary material).

All tests of statistical hypothesis were made on the two-sided 5% level of significance. No adjustment for multiplicity of statistical tests was made.

## Results

The study population of 1-year survivors comprised of 5,582,360 individuals. We excluded 4171 (0.1%) with zero 5-min Apgar score and an additional 59,338 (1.1%) with missing 5 min-Apgar score. Of the remaining 5,518,851 individuals, 184,076 (3.3%) had missing data on one or more covariates yielding 5,341,203 subjects (Denmark, 1,480,707; Norway, 1,130,453; Sweden, 2,375,502; and Western Australia, 354,541) included for analysis. These included 8,430 (0.16%) individuals with low (Apgar score 1–3), 39,234 (0.73%) individuals with intermediate (Apgar score 4–6), and 5,293,539 (99.11%) individuals with optimal Apgar score (Apgar score 7–10). There were 33,244 children with ASD (0.62%) and 11,543 (0.22%) with AD specifically. Table 1 summarizes distribution of cases and covariates by Apgar score categories.

**Table 1:** Covariate distribution by Apgar score

Variables	Apgar score		
	Low (1–3)	Intermediate (4–6)	Optimal (7–10)
Birth (N)	8430	39,234	5,293,539
ASD [N (%)]	92 (1.09)	409 (1.04)	32,743 (0.62)
AD only [N (%)]	46 (0.55)	161 (0.41)	11,336 (0.21)
Birth year, intervals [N (%)]			
1984–1988	2006 (23.80)	8092 (20.62)	1,069,112 (20.20)
1989–1993	1908 (22.63)	8836 (22.52)	1,250,702 (23.63)
1994–1998	1840 (21.83)	8384 (21.37)	1,160,123 (21.92)
1999–2003	1520 (18.03)	8319 (21.20)	1,058,272 (19.99)
2004–2007	1156 (13.71)	5603 (14.28)	755,330 (14.27)
Site [N (%)]			
Denmark	1313 (15.58)	7411 (18.89)	1,471,983 (27.81)
Norway	1396 (16.56)	8717 (22.22)	1,120,340 (21.16)
Sweden	5277 (62.60)	18,915 (48.12)	2,351,310 (44.42)
Western Australia	444 (5.27)	4191 (10.68)	349,906 (6.61)
Sex, Female [N (%)]	3828 (45.41)	16,810 (42.85)	2,577,784 (48.70)
Maternal age, intervals, years [N (%)]			
≤ 19	204 (2.42)	1005 (2.56)	109,313 (2.07)
20–24	1449 (17.19)	6966 (17.76)	906,409 (17.12)
25–29	2845 (33.75)	13,542 (34.52)	1,896,874 (35.83)
30–34	2509 (29.76)	11,434 (29.14)	1,614,498 (30.50)
35–39	1175 (13.94)	5180 (13.20)	648,005 (12.24)
≥ 40	248 (2.94)	1107 (2.82)	118,440 (2.24)
Paternal age, intervals, years [N (%)]			
≤ 19	65 (0.77)	252 (0.64)	28,253 (0.53)
20–24	756 (8.97)	3841 (9.79)	448,661 (8.48)
25–29	2329 (27.63)	11,101 (28.29)	1,481,302 (27.98)
30–34	2676 (31.74)	12,445 (31.72)	1,784,391 (33.71)
35–39	1614 (19.15)	7257 (18.50)	1,020,575 (19.28)
40–44	671 (7.96)	2935 (7.48)	373,821 (7.06)
≥ 45	319 (3.78)	1403 (3.58)	156,536 (2.96)
Gestational age, weeks [N (%)]			
≤ 36	2505 (29.72)	10,247 (26.12)	306,734 (5.79)
37–40	3982 (47.24)	18,813 (47.95)	3,616,704 (68.32)
≥ 41	1943 (23.05)	10,174 (25.93)	1,370,101 (25.88)
Birth weight, g [N (%)]			
< 1000	640 (7.59)	1906 (4.86)	7624 (0.14)
1000–1499	486 (5.77)	1891 (4.82)	21,564 (0.41)
1500–2499	1111 (13.18)	5169 (13.17)	196,433 (3.71)
2500–3999	4972 (58.98)	24,065 (61.34)	4,095,304 (77.36)
≥ 4000	1221 (14.48)	6203 (15.81)	972,614 (18.37)

*ASD* autism spectrum disorder, *N* number

### Association between three categories of Apgar score and ASD

In model 1, low (RR, 1.76; 95% CI, 1.44–2.16) and intermediate (RR, 1.84; 95% CI, 1.67–2.02) Apgar scores were associated with a higher RR of ASD compared to optimal Apgar score in the basic model. Adjusting for confounders (models 2–4) reduced the RR estimates by about 20% in the fully adjusted model mostly after also adjusting for gestational age and birth weight (models 3 and 4). Restricting the analyses to singleton births had only minimal effects in the estimates of RR (Table 2).

**Table 2:** Risk of ASD by Apgar score

Apgar score	Prevalence cases/number of children (%)	All births, RR (95% CI)			
		Model 1^a^	Model 2^b^	Model 3^c^	Model 4^d^
N = 5,341,203					
1–3	92/8430 (1.09)	1.76 (1.44–2.16)	1.70 (1.39–2.09)	1.57 (1.29–1.93)	1.42 (1.16–1.74)
4–6	409/39,234 (1.04)	1.84 (1.67–2.02)	1.72 (1.56–1.90)	1.61 (1.46–1.78)	1.50 (1.36–1.65)
7–10 (ref)	32,743/5293,539 (0.62)	1.00	1.00	1.00	1.00

Apgar score	Prevalence cases/number of children (%)	Singleton births only RR (95% CI)			
		Model 1	Model 2	Model 3	Model 4
N = 5,196,250					
1–3	86/7769 (1.11)	1.79 (1.45–2.21)	1.72 (1.39–2.12)	1.58 (1.28–1.95)	1.43 (1.15–1.76)
4–6	385/36,204 (1.06)	1.88 (1.70–2.07)	1.76 (1.59–1.94)	1.65 (1.49–1.82)	1.53 (1.38–1.69)
7–10 (ref)	31,812/5144,511 (0.62)	1.00	1.00	1.00	1.00

The estimates are RR (95% CI)

*ASD* Autism spectrum disorder, *N* number, *RR* relative risk, *95% CI* 95% confidence intervals

^a^Adjusted for site and birth year

^b^Adjusted for site, birth year, and maternal and paternal age, sex

^c^Adjusted for site, birth year, and maternal and paternal age, sex, and gestational age

^d^Adjusted for site, birth year, and maternal and paternal age, sex, gestational age, and birth weight

### Association between individual Apgar scores and ASD

**Fig. 1:**
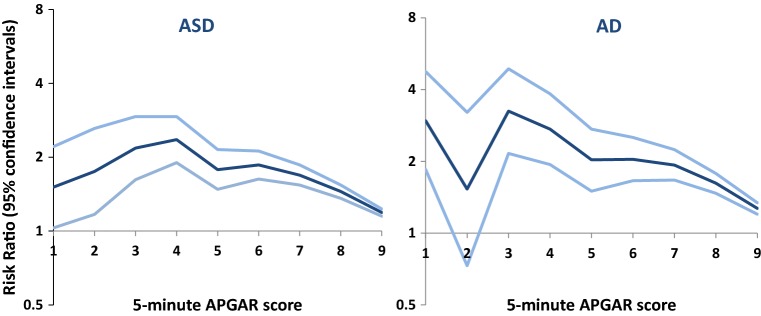
Risk ratio and 95% confidence intervals for the association between individual Apgar scores and autism spectrum disorder (ASD) and autistic disorder (AD). Reference group is Apgar score of 10

From graphical evaluation (Fig. 1), there risk for ASD was almost unchanged from Apgar score 1–4, and decreased risk thereafter in an almost linear fashion with increasing Apgar scores. This reverse U shape relationship was supported by a statistically significant quadratic term in fully adjusted models (*p* value for the quadratic term < 0.001).

### Effect of sex

Stratification by sex indicated a slightly higher risk associated with low and intermediate Apgar score in females compared with males (Table 3). The interaction term was statistically significant in the fully adjusted model, showing that the slope of the association between Apgar score and risk of ASD was statistically significantly steeper for females than males. *p* value for the Wald’s test of interaction in fully adjusted model was 0.014).

**Table 3:** Risk of ASD by Apgar score stratified by sex

Apgar score	Prevalence cases/number of children (%)	Females, RR (95% CI)		
		Model 1^a^	Model 2^b^	Model 3^c^
N = 2,602,333				
1–3	30/3828 (0.78)	2.28 (1.59–3.25)	2.02 (1.42–2.89)	1.78 (1.24–2.56)
4–6	100/16,710 (0.59)	2.05 (1.68–2.50)	1.84 (1.51–2.24)	1.65 (1.35–2.02)
7–10 (ref)	8068/2,569,716 (0.31)	1.00	1.00	1.00

Apgar score	Prevalence cases/number of children (%)	Males, RR (95% CI)		
		Model 1	Model 2	Model 3
N = 2,747,017				
1–3	62/4602 (1.35)	1.54 (1.20–1.97)	1.43 (1.11–1.83)	1.30 (1.01–1.66)
4–6	309/22,424 (1.38)	1.66 (1.48–1.85)	1.56 (1.39–1.74)	1.46 (1.30–1.63)
7–10 (ref)	24,675/2,715,755 (0.91)	1.00	1.00	1.00

The estimates are RR (95% CI)

*ASD* Autism spectrum disorder, *N* number, *RR* relative risk, *95% CI* 95% confidence intervals

^a^Adjusted for site and birth year

^b^Adjusted for site, birth year, and maternal and paternal age, and gestational age

^c^Adjusted for site, birth year, and maternal and paternal age, gestational age, and birth weight

### Effect of gestational age

There was modest support for effect-modification by gestational age on the association between Apgar score and ASD in all but fully adjusted models (Table 4). Importantly, among strata of gestational age, only in preterm infants did birth weight show a marked confounding effect, reducing the RRs by more than 20% (Table 4). However after adjusting for birth weight, the Wald’s test for interaction lost statistical significance (*p* value = 0.353 for ASD).

**Table 4:** Risk of ASD by Apgar score stratified by gestational age

Apgar score	Prevalence cases/number of children (%)	≤ 36 weeks, RR (95% CI)		
		Model 1^a^	Model 2^b^	Model 3^c^
N = 320,916				
1–3	32/2505 (1.28)	1.48 (1.05–2.02)	1.45 (1.02–2.04)	1.14 (0.80–1.62)
4–6	156/10,247 (1.52)	1.96 (1.67–2.30)	1.90 (1.62–2.23)	1.58 (1.33–1.86)
7–10 (ref)	2481/306,734 (0.81)	1.00	1.00	1.00

Apgar score	Prevalence cases/number of children (%)	37–40 weeks, RR (95% CI)		
		Model 1	Model 2	Model 3
N = 3,644,332				
1–3	36/3982 (0.90)	1.50 (1.09–2.08)	1.56 (1.14–2.13)	1.53 (1.12–2.09)
4–6	162/18,813 (0.86)	1.56 (1.34–1.82)	1.53 (1.32–1.79)	1.44 (1.24–1.68)
7–10 (ref)	21,867/3,616,704 (0.60)	1.00	1.00	1.00

Apgar score	Prevalence cases/number of children (%)	≥ 41 weeks, RR (95% CI)		
		Model 1	Model 2	Model 3
N = 1,384,102				
1–3	24/1943 (1.24)	2.00 (1.34–2.97)	1.95 (1.31–2.90)	1.94 (1.30–2.88)
4–6	91/10,174 (0.89)	1.56 (1.27–1.92)	1.46 (1.19–1.79)	1.45 (1.18–1.78)
7–10 (ref)	8395/1,370,101 (0.61)	1.00	1.00	1.00

The estimates are RR (95% CI)

*ASD* Autism spectrum disorder, *N* number, *RR* relative risk, *95% CI* 95% confidence intervals

^a^Adjusted for site and birth year

^b^Adjusted for site, birth year, and maternal and paternal age, and sex

^c^Adjusted for site, birth year, and maternal and paternal age, sex, and birth weight

### Supplementary results: analysis of AD

In model 1, we found RR of 2.54 (95% CI, 1.91–3.38) and RR of 2.00 (95% CI, 1.72–2.33) for AD for low and intermediate Apgar scores respectively (Supplementary Table 1), whereas in fully adjusted models the estimates were (RR, 1.88; 95% CI, 1.41–2.51) and (RR, 1.54; 95% CI, 1.32–1.81) respectively (Supplementary Tables 1–3). In analysis of continuous Apgar scores, while the point estimates for AD were slightly larger than for ASD (although the 95% CI overlap), the RR pattern was similar for ASD and AD. The quadratic term for Apgar score in fully adjusted models (*p* value for the quadratic term < 0.001) was significant. In fully adjusted models, the interaction term for sex and Apgar score was statistically significant (*p* value for Wald’s test = 0.004), whereas the interaction term for gestational age and Apgar score was not significant (*p* value for Wald’s test = 0.260).

## Discussion

Using the largest multinational population-based ASD database to investigate the association between 5-min Apgar score and ASD, we found a 75–80% increase in risk of ASD in neonates with low to intermediate Apgar score compared with neonates with optimal Apgar score. Adjusting for the potential confounding effects of gestational age and birth weight) reduced the magnitude of the RRs for the different categories compared to the least adjusted models by about 20%. Nevertheless, the association between Apgar score and risk of ASD remained statistically significantly larger than one.

Although several previous studies investigated the association between Apgar score and ASD, we took advantage of the large data source available from iCARE to study this association in greater depth [31]. Specifically, we investigated the effect of two diagnostic definitions (ASD and AD) as well as two potentially important modifiers, namely sex and gestational age and one potentially important confounder birth weight, on the association between Apgar score and ASD.

An interaction term for Apgar score and sex was statistically significant, indicating that effect of low Apgar score on risk of ASD depends on sex, with girls with low Apgar score having a higher RR for ASD than boys. Of note, this does not mean that boys with low Apgar score are at lower risk for ASD than girls, but rather signifies a larger role for low Apgar score in risk of ASD in girls compared with boys. Given that Apgar score is an indication of neonatal vitality, it can be inferred that factors that reduce infant vitality contribute slightly more to risk of ASD in girls than in boys. This finding might explain a part of higher frequency of epilepsy [37] and intellectual disability [27] (other factors related to reduced vitality [30, 38]) in girls with ASD.

In general, RR estimates for low Apgar score were reduced after adjusting for gestational age and birth weight, raising the possibility that both might be confounders. Of note, in preterm infants, the confounding effect of birth weight on the association between Apgar score and risk for ASD was strong. This is in line with the existing evidence, because preterm and low birth weight infants are less mature and therefore often more likely to present with lower Apgar scores than term infants [39, 40]. In fact, several studies have shown that low Apgar score is less predictive of unfavorable outcomes in preterm infants than in term infants [17, 39–41].

The fact that there was little change in RR of ASD from Apgar scores of 1–4 (Fig. 1), which may seem counterintuitive, could be due to “diagnostic overshadowing” [42, 43]. Many children with extremely low Apgar scores experience multiple severe outcomes (including CP or severe ID) that might preclude diagnosis of ASD.

Several potential mechanisms may underlie the observed association between low Apgar score and AD. First, the association might reflect a shared genetic background between factors that reduce newborn vitality and ASD. Second, the association between Apgar score and ASD may be a marker of the underlying ASD phenotype, that is, Apgar as a marker of fetal status may reflect a process set in motion by the underlying mechanism of and/or comorbidities of ASD at some earlier point in pregnancy. For example, risk for several congenital anomalies is increased in patients with ASD [44]; many of these anomalies are associated with poorer neonatal outcomes and therefore lower Apgar score at birth [45]. Similarly, intrauterine infections can cause abnormal neurodevelopment (increasing the risk of ASD) [46] while at the same time causing cardiorespiratory anomalies leading to a lower Apgar score [47]. Importantly, Apgar score alone is an insensitive tool for defining the process culminating in an ASD diagnosis. Third, given that ASD is largely a multifactorial condition, this association might reflect the effect of environmental factors (i.e. birth complications, hypoxia) on risk of ASD [3].

Our study had some limitations. Despite the large sample size, unfortunately the data on socioeconomic status, psychiatric comorbidity (e.g. ADHD, epilepsy or ID) and parental medical and psychiatric history were unavailable. Socioeconomic status (SES) is considered an important determinant of health and could potentially affect the association between low Apgar score and ASD in various ways and this association could possibly change over time. Whereas a large population-based study from Denmark found no significant association between SES and ASD [48], a Swedish study has shown an association between lower family income and higher risk for ASD [49]. Although the sites included in the present study have policies that provide equal healthcare to all regardless of their SES, they are not immune to the SES-related diagnostic bias. The evidence on the association between socioeconomic status and Apgar score remains inconsistent but does suggest a trend towards worse Apgar score in families with lower SES [13, 50]. However, whether a low Apgar score (or related problems) changes the likelihood of the diagnosis is uncertain. Data on psychiatric and neurological comorbidities could have been potentially useful for investigating the specificity of the observed association between Apgar score and risk of ASD. For example, ID, CP and ADHD are all associated with low Apgar score, with magnitudes being the highest for the association between low Apgar score and CP or ID [16, 25, 51, 52]. This differential association might be due to differences in the severity of underlying pathophysiology, different biological mechanisms, or merely different underlying genetic risk associated with each of these conditions. Maternal psychiatric conditions such as depression and anxiety and maternal antidepressant use and maternal medical conditions such as diabetes mellitus and autoimmune diseases have also been variably linked to ASD [3, 53, 54]. Even though maternal smoking has been associated with low Apgar score [55], there is little evidence for its association with the risk for ASD [56]. The same conditions have been associated with low Apgar score at birth in some [57, 58] but not all studies [59]. Furthermore, studying severity of autistic symptoms and autistic subtypes in more details could have given us a better understanding of the observed association between low Apgar score and risk for ASD, although analyzing AD cases (which are considered the more severe end of the ASD) separately might fulfill that goal to some extent.

## Conclusions

Low Apgar score is associated with higher risk of ASD. We did not observe any major modifying effects of gestational age and sex, although birth weight and gestational both act as confounders. Despite an increased relative risk of ASD/AD in children with low Apgar score, the absolute risk is very small. Therefore, even if Apgar score is a marker of an unknown causal factor(s), the associated risk signifies only a minor contribution to the overall burden of ASD/AD. Future studies should expand this multi cohort approach to other neurodevelopmental outcomes such as ADHD and ID.

## Electronic supplementary material

Below is the link to the electronic supplementary material.

Supplementary material 1 (PDF 164 kb)

## References

[1] De Rubeis S (2014). Synaptic, transcriptional and chromatin genes disrupted in autism. Nature.

[2] Chaste P, Leboyer M (2012). Autism risk factors: genes, environment, and gene–environment interactions. Dialogues Clin Neurosci.

[3] Modabbernia A, Velthorst E, Reichenberg A (2017). Environmental risk factors for autism: an evidence-based review of systematic reviews and meta-analyses. Mol Autism.

[4] Thorngren-Jerneck K, Herbst A (2001). Low 5-minute Apgar score: a population-based register study of 1 million term births. Obstet Gynecol.

[5] Burd L (1999). Prenatal and perinatal risk factors for autism. J Perinat Med.

[6] Dodds L (2011). The role of prenatal, obstetric and neonatal factors in the development of autism. J Autism Dev Disord.

[7] Polo-Kantola P (2014). Obstetric risk factors and autism spectrum disorders in Finland. J Pediatr.

[8] Gardener H, Spiegelman D, Buka SL (2009). Prenatal risk factors for autism: comprehensive meta-analysis. Br J Psychiatry.

[9] Wang C (2017). Prenatal, perinatal, and postnatal factors associated with autism: a meta-analysis. Medicine (Baltimore).

[10] American Academy Of Pediatrics Committee on Fetus and Newborn (2015). The Apgar score. Pediatrics.

[11] Iliodromiti S (2014). Apgar score and the risk of cause-specific infant mortality: a population-based cohort study. Lancet.

[12] Hogan L (2007). How often is a low 5-min Apgar score in term newborns due to asphyxia?. Eur J Obstet Gynecol Reprod Biol.

[13] Lai S, Flatley C, Kumar S (2017). Perinatal risk factors for low and moderate five-minute Apgar scores at term. Eur J Obstet Gynecol Reprod Biol.

[14] Apgar V (1953). A proposal for a new method of evaluation of the newborn infant. Curr Res Anesth Analg.

[15] O’Donnell CP (2006). Interobserver variability of the 5-minute Apgar score. J Pediatr.

[16] Moster D (2001). The association of Apgar score with subsequent death and cerebral palsy: a population-based study in term infants. J Pediatr.

[17] Casey BM, McIntire DD, Leveno KJ (2001). The continuing value of the Apgar score for the assessment of newborn infants. N Engl J Med.

[18] Drage JS (1966). The Apgar score as an index of infant morbidity. A report from the collaborative study of cerebral palsy. Dev Med Child Neurol.

[19] Persson M (2018). Five and 10 minute Apgar scores and risks of cerebral palsy and epilepsy: population based cohort study in Sweden. BMJ.

[20] Buchmayer S (2009). Can association between preterm birth and autism be explained by maternal or neonatal morbidity?. Pediatrics.

[21] Mrozek-Budzyn D, Majewska R, Kieltyka A (2013). Prenatal, perinatal and neonatal risk factors for autism-study in Poland. Cent Eur J Med.

[22] Mason-Brothers A (1990). The UCLA-University of Utah epidemiologic survey of autism: prenatal, perinatal, and postnatal factors. Pediatrics.

[23] Bilder D (2009). Prenatal, perinatal, and neonatal factors associated with autism spectrum disorders. Pediatrics.

[24] Burstyn I, Sithole F, Zwaigenbaum L (2010). Autism spectrum disorders, maternal characteristics and obstetric complications among singletons born in Alberta, Canada. Chronic Dis Can.

[25] Modabbernia A (2016). Impaired gas exchange at birth and risk of intellectual disability and autism: a meta-analysis. J Autism Dev Disord.

[26] Van Wijngaarden-Cremers PJ (2014). Gender and age differences in the core triad of impairments in autism spectrum disorders: a systematic review and meta-analysis. J Autism Dev Disord.

[27] Rivet TT, Matson JL (2011). Review of gender differences in core symptomatology in autism spectrum disorders. Res Autism Spectr Disord.

[28] Saigal S, Doyle LW (2008). An overview of mortality and sequelae of preterm birth from infancy to adulthood. Lancet.

[29] Bekedam DJ (2002). Male predominance in fetal distress during labor. Am J Obstet Gynecol.

[30] Sun Y (2006). Apgar scores and long-term risk of epilepsy. Epidemiology.

[31] Schendel DE (2013). The International Collaboration for Autism Registry Epidemiology (iCARE): multinational registry-based investigations of autism risk factors and trends. J Autism Dev Disord.

[32] Sun Y (2006). Paternal age and Apgar scores of newborn infants. Epidemiology.

[33] Vestergaard M (2008). Death in children with febrile seizures: a population-based cohort study. Lancet.

[34] Petersen DJ (2006). The population prevalence of child psychiatric disorders in Danish 8- to 9-year-old children. Eur Child Adolesc Psychiatry.

[35] Suren P (2012). Autism spectrum disorder, ADHD, epilepsy, and cerebral palsy in Norwegian children. Pediatrics.

[36] Sandin S (2015). Autism risk associated with parental age and with increasing difference in age between the parents. Mol Psychiatry.

[37] Amiet C (2008). Epilepsy in autism is associated with intellectual disability and gender: evidence from a meta-analysis. Biol Psychiatry.

[38] Bilder DA (2013). Prenatal and perinatal factors associated with intellectual disability. Am J Intellect Dev Disabil.

[39] Catlin EA (1986). The Apgar score revisited: influence of gestational age. J Pediatr.

[40] Hegyi T (1998). The apgar score and its components in the preterm infant. Pediatrics.

[41] Jensen LV (2012). Low 5-min Apgar score in moderately preterm infants; association with subsequent death and cerebral palsy: a register based Danish national study. Acta Paediatr.

[42] Jopp DA, Keys CB (2001). Diagnostic overshadowing reviewed and reconsidered. Am J Ment Retard.

[43] Reiss S, Szyszko J (1983). Diagnostic overshadowing and professional experience with mentally retarded persons. Am J Ment Defic.

[44] Wier ML (2006). Congenital anomalies associated with autism spectrum disorders. Dev Med Child Neurol.

[45] Clausson B, Cnattingius S, Axelsson O (1999). Outcomes of post-term births: the role of fetal growth restriction and malformations. Obstet Gynecol.

[46] Gardener H, Spiegelman D, Buka SL (2011). Perinatal and neonatal risk factors for autism: a comprehensive meta-analysis. Pediatrics.

[47] Garnier Y (2003). Infection-related perinatal brain injury: the pathogenic role of impaired fetal cardiovascular control. J Soc Gynecol Investig.

[48] Larsson HJ (2005). Risk factors for autism: perinatal factors, parental psychiatric history, and socioeconomic status. Am J Epidemiol.

[49] Rai D (2012). Parental socioeconomic status and risk of offspring autism spectrum disorders in a Swedish population-based study. J Am Acad Child Adolesc Psychiatry.

[50] Odd DE (2008). Risk of low Apgar score and socioeconomic position: a study of Swedish male births. Acta Paediatr.

[51] Zhu T (2016). Association between perinatal hypoxic-ischemic conditions and attention-deficit/hyperactivity disorder: a meta-analysis. J Child Neurol.

[52] Kotlicka-Antczak M (2014). Obstetrical complications and Apgar score in subjects at risk of psychosis. J Psychiatr Res.

[53] Mezzacappa A (2017). Risk for autism spectrum disorders according to period of prenatal antidepressant exposure: a systematic review and meta-analysis. JAMA Pediatr.

[54] Daniels JL (2008). Parental psychiatric disorders associated with autism spectrum disorders in the offspring. Pediatrics.

[55] Straube S (2010). Investigation of the association of Apgar score with maternal socio-economic and biological factors: an analysis of German perinatal statistics. Arch Gynecol Obstet.

[56] Rosen BN (2015). Maternal smoking and autism spectrum disorder: a meta-analysis. J Autism Dev Disord.

[57] Berle JO (2005). Neonatal outcomes in offspring of women with anxiety and depression during pregnancy. A linkage study from The Nord-Trondelag Health Study (HUNT) and Medical Birth Registry of Norway. Arch Womens Ment Health.

[58] Simon GE, Cunningham ML, Davis RL (2002). Outcomes of prenatal antidepressant exposure. Am J Psychiatry.

[59] Andersson L (2004). Neonatal outcome following maternal antenatal depression and anxiety: a population-based study. Am J Epidemiol.

