# Supplementary material for: Apgar score and risk of autism
Source: Eur J Epidemiol. 2018 Oct 5;34(2):105–14. doi: 10.1007/s10654-018-0445-1 (PMC6373297; doi:10.1007/s10654-018-0445-1)
Supplement: Supplementary file 1 — Supplementary material 1 (PDF 164 kb) [file 10654_2018_445_MOESM1_ESM.pdf]

Supplementary Table 1. Risk of AD by Apgar score

Disorder	Apgar score	Prevalence cases/number of children (%)	All Births, RR (95% CI)			
**AD (N=5,319,642)**			**Model 1[a]**	**Model 2[b]**	**Model 3[c]**	**Model 4[d]**
	**1-3**	46/8,385 (0.55)	2.54 (1.91-3.38)	2.44 (1.84-3.25)	2.20 (1.65-2.93)	1.88 (1.41-2.51)
	**4-6**	161/38,990 (0.41)	2.00 (1.72-2.33)	1.87 (1.61-2.18)	1.72 (1.47-2.01)	1.54 (1.32-1.81)
	**7-10 (ref)**	11,336/5,272,267 (0.21)	1.00	1.00	1.00	1.00
			**Singleton Births only RR (95% CI)**			
**AD (N=5,175,194)**			**Model 1**	**Model 2**	**Model 3**	**Model 4**
	**1-3**	43/7,727 (0.56)	2.60 (1.93-3.49)	2.57 (1.92-3.46)	2.24 (1.66-3.01)	1.89 (1.41-2.55)
	**4-6**	150/ 35973 (0.41)	2.04 (1.74-2.39)	2.03 (1.73-2.38)	1.76 (1.50-2.06)	1.58 (1.34-1.85)
	**7-10 (ref)**	10,941/5,123,772 (0.21)	1.00	1.00	1.00	1.00

Abbreviations: ASD: Autism Spectrum Disorder; AD: Autistic Disorder; N: Number; RR: Relative Risk; 95%CI: 95% confidence intervals

The estimates are RR (95% CI)

a. Adjusted for site and birth year

b. Adjusted for site, birth year, and maternal and paternal age, sex

c. Adjusted for site, birth year, and maternal and paternal age, sex, and gestational age

d. Adjusted for site, birth year, and maternal and paternal age, sex, gestational age, and birth weight

Supplementary Table 2. Risk of AD by Apgar score stratified by sex

Disorder	Apgar score	Prevalence cases/ number of children (%)	Females, RR (95% CI)		
**AD (N=2,596,800)**			**Model 1**	**Model 2**	**Model 3**
	**1-3**	15/3,814 (0.39)	3.74 (2.29-6.11)	3.20 (1.96-5.23)	2.62 (1.59-4.32)
	**4-6**	41/16,755 (0.24)	2.70 (2.01-3.63)	2.35 (1.75-3.16)	2.01 (1.49-2.73)
	**7-10 (ref)**	2,517/2,592,851 (0.10)	1.00	1.00	1.00
			**Males, RR (95%CI)**		
**AD (N=2,730,942)**			**Model 1**	**Model 2**	**Model 3**
	**1-3**	31/4,571 (0.68)	2.10 (1.48-2.98)	1.90 (1.34-2.70)	1.65 (1.16-2.35)
	**4-6**	120/22,235 (0.54)	1.69 (1.41-2.02)	1.57 (1.31-1.88)	1.43 (1.19-1.71)
	**7-10 (ref)**	8,819/2,699,929 (0.33)	1.00	1.00	1.00

Abbreviations: ASD: Autism Spectrum Disorder; AD: Autistic Disorder; N: Number; RR: Relative Risk; 95%CI: 95% confidence intervals
The estimates are RR (95%CI)
a. Adjusted for site and birth year
b. Adjusted for site, birth year, and maternal and paternal age, and gestational age
c. Adjusted for site, birth year, and maternal and paternal age, gestational age, and birth weight

Table 3. Risk of AD by Apgar score stratified by gestational age

Disorder	Apgar score	Prevalence cases/number of children (%)	≤36 weeks, RR (95%CI)		
**AD (N=319,284)**			**Model 1**	**Model 2**	**Model 3**
	**1-3**	21/2,494 (0.84)	2.56 (1.67-3.95)	2.48 (1.61-3.82)	1.80 (1.16-2.80)
	**4-6**	75/10,168 (0.74)	2.49 (1.97-3.14)	2.41 (1.91-3.04)	1.88 (1.47-2.39)
	**7-10 (ref)**	938/305,201 (0.31)	1.00	1.00	1.00
			**37-40 weeks, RR (95% CI)**		
**AD (N=3,629,958)**			**Model 1**	**Model 2**	**Model 3**
	**1-3**	14/3,961 (0.35)	1.77 (1.07-2.94)	1.71 (1.03-2.84)	1.65 (0.99-2.73)
	**4-6**	57/18,710 (0.30)	1.52 (1.18-1.97)	1.43 (1.11-1.84)	1.38 (1.07-1.79)
	**7-10 (ref)**	7,562/3,602,491 (0.21)	1.00	1.00	1.00
			**≥41 weeks, RR (95% CI)**		
**AD (N=1,378,490)**			**Model 1**	**Model 2**	**Model 3**
	**1-3**	11/1,930 (0.57)	2.62 (1.45-4.72)	2.57 (1.42-4.63)	2.53 (1.40-4.56)
	**4-6**	29/10,112 (0.29)	1.36 (0.95-1.97)	1.27 (0.88-1.83)	1.26 (0.87-1.82)
	**7-10 (ref)**	2,836/1,364,575 (0.21)	1.00	1.00	1.00

Abbreviations: ASD: Autism Spectrum Disorder; AD: Autistic Disorder; N: Number; RR: Relative Risk; 95%CI: 95% confidence intervals
The estimates are RR (95%CI)
a. Adjusted for site and birth year
b. Adjusted for site, birth year, and maternal and paternal age, and sex
c. Adjusted for site, birth year, and maternal and paternal age, sex, and birth weight
